# Pixel-wise statistical analysis of myocardial injury in STEMI patients with delayed enhancement MRI

**DOI:** 10.3389/fcvm.2023.1136760

**Published:** 2023-06-16

**Authors:** Nicolas Duchateau, Magalie Viallon, Lorena Petrusca, Patrick Clarysse, Nathan Mewton, Loic Belle, Pierre Croisille

**Affiliations:** ^1^ Univ Lyon, CREATIS, INSA, CNRS UMR 5220, INSERM U1294, Université Lyon 1, UJM Saint-Etienne, Lyon, France; ^2^ Institut Universitaire de France (IUF), Paris, France; ^3^ Department of Radiology, Hôpital Nord, University Hospital of Saint-Étienne, Saint-Étienne, France; ^4^ Department of Cardiology, Clinical Investigation Center, INSERM 1407, Hôpital Cardiovasculaire Louis Pradel, Lyon, France; ^5^ Department of Cardiology, Centre Hospitalier Annecy-Genevois, Annecy, France

**Keywords:** acute myocadial infarction, delayed enhancement MRI, statistical atlas, infarct size, microvascular obstruction

## Abstract

**Objectives:**

Myocardial injury assessment from delayed enhancement magnetic resonance images is routinely limited to global descriptors such as size and transmurality. Statistical tools from computational anatomy can drastically improve this characterization, and refine the assessment of therapeutic procedures aiming at infarct size reduction. Based on these techniques, we propose a new characterization of myocardial injury up to the pixel resolution. We demonstrate it on the imaging data from the Minimalist Immediate Mechanical Intervention randomized clinical trial (MIMI: NCT01360242), which aimed at comparing immediate and delayed stenting in acute ST-Elevation Myocardial Infarction (STEMI) patients.

**Methods:**

We analyzed 123 patients from the MIMI trial (62 ± 12 years, 98 male, 65 immediate 58 delayed stenting). Early and late enhancement images were transported onto a common geometry using techniques inspired by statistical atlases, allowing pixel-wise comparisons across population subgroups. A practical visualization of lesion patterns against specific clinical and therapeutic characteristics was also proposed using state-of-the-art dimensionality reduction.

**Results:**

Infarct patterns were roughly comparable between the two treatments across the whole myocardium. Subtle but significant local differences were observed for the LCX and RCA territories with higher transmurality for delayed stenting at lateral and inferior/inferoseptal locations, respectively (15% and 23% of myocardial locations with a *p*-value <0.05, mainly in these regions). In contrast, global measurements were comparable for all territories (no statistically significant differences for all-except-one measurements before standardization / for all after standardization), although immediate stenting resulted in more subjects without reperfusion injury.

**Conclusion:**

Our approach substantially empowers the analysis of lesion patterns with standardized comparisons up to the pixel resolution, and may reveal subtle differences not accessible with global observations. On the MIMI trial data as illustrative case, it confirmed its general conclusions regarding the lack of benefit of delayed stenting, but revealed subgroups differences thanks to the standardized and finer analysis scale.

## 1. Introduction

Cardiac magnetic resonance imaging (MRI) has a central role in myocardial infarction experimental and clinical trials, and is frequently recognized as providing the best *in vivo* surrogate endpoints for infarct size and microvascular obstruction (MVO) (1–3). The lesion patterns visible on delayed enhancement images vary from ischemia-reperfusion lesions to final scar within the infarct zone on late Gadolinium enhancement (LGE), with or without MVO region on early Gadolinium enhancement (EGE) and LGE and at the acute phase. However, most analyses only assess a tiny part of the imaging data related to these lesions, whose distribution within the myocardium and across slices is complex. They focus on simple global measurements (mainly size, transmurality, and endocardial surface area) (4–6), which drastically limit the quantification of the mechanisms of ischemia-reperfusion and treatment effects. Recent tools (not limited to MRI) offer more detailed assessment and in particular standardized anatomical diagrams (7), but are still insufficiently exploited for the analysis of populations.

Computational tools inspired by statistical atlases can overcome the main bottleneck to a finer analysis of such lesions, namely anatomical differences across subjects and acquisitions (8). First, they allow advanced statistical analysis of myocardial shape differences across a population, as recently demonstrated on acute myocardial infarction patients (9). In addition, as in our study, they allow bringing all the imaging data to a standardized geometry (the “atlas”). This means that much finer statistical comparisons can be performed, up to examining differences in the lesion patterns at each pixel of the myocardium in the EGE and LGE images. This operation is a prerequisite to detect potential inter-subject differences inaccessible and not evaluated by global or regional descriptors of the lesions. In addition, in a logic of personalized medicine, these tools allow understanding and visualizing how a subject is positioned with respect to a given population and/or how she/he responds to a given therapeutic intervention.

In this paper, we re-examined the delayed enhancement images from the Minimalist Immediate Mechanical Intervention (MIMI) trial (10) with such techniques. This multicenter randomized clinical trial aimed at comparing immediate and delayed stenting in acute ST-Elevation Myocardial Infarction (STEMI) patients, two treatment strategies that are still under debate (11, 12). By examining commonly-used global descriptors of the lesions, the original MIMI study failed to support delayed stenting against immediate stenting, and even suggested a deleterious effect of delayed stenting on MVO size. However, similar studies reported potential benefits of such intervention regarding infarct size or the incidence of MVO (13, 14). Thus, it sounds as a relevant playground to evaluate if a finer analysis, with standardized comparisons up to the pixel resolution, could better characterize infarct and MVO pattern differences across subjects and provide a fresh look at the quantification of the intervention outcome.

## 2. Materials and methods

### 2.1. Participants

This study retrospectively analyzed the imaging data from the MIMI study (ClinicalTrials ID: NCT01360242), which was a multicenter, prospective, randomized, open-label trial with blinded endpoint evaluation. The study protocol was approved by the local ethics committee (IRB 2010–048), and complied with the Declaration of Helsinki and French laws. All subjects gave written informed consent.

The included patients were adults with symptoms consistent with STEMI ≤12 h, with ST-segment-elevation ≥1 mm in ≥2 contiguous limb leads or >2 mm in ≥2 precordial leads on the electrocardiogram, intended for primary percutaneous coronary intervention. The clinical and angiographic inclusion criteria and the intervention procedure were detailed in Belle et al. (10).

Patients were randomized between two different strategies: immediate stenting or 24–48 h delayed stenting. The primary endpoint was MVO [% of left ventricular (LV) mass] on cardiac magnetic resonance performed 5 days (interquartile range 4–6) after inclusion.

### 2.2. Imaging protocol and labeling

**Figure 1:**
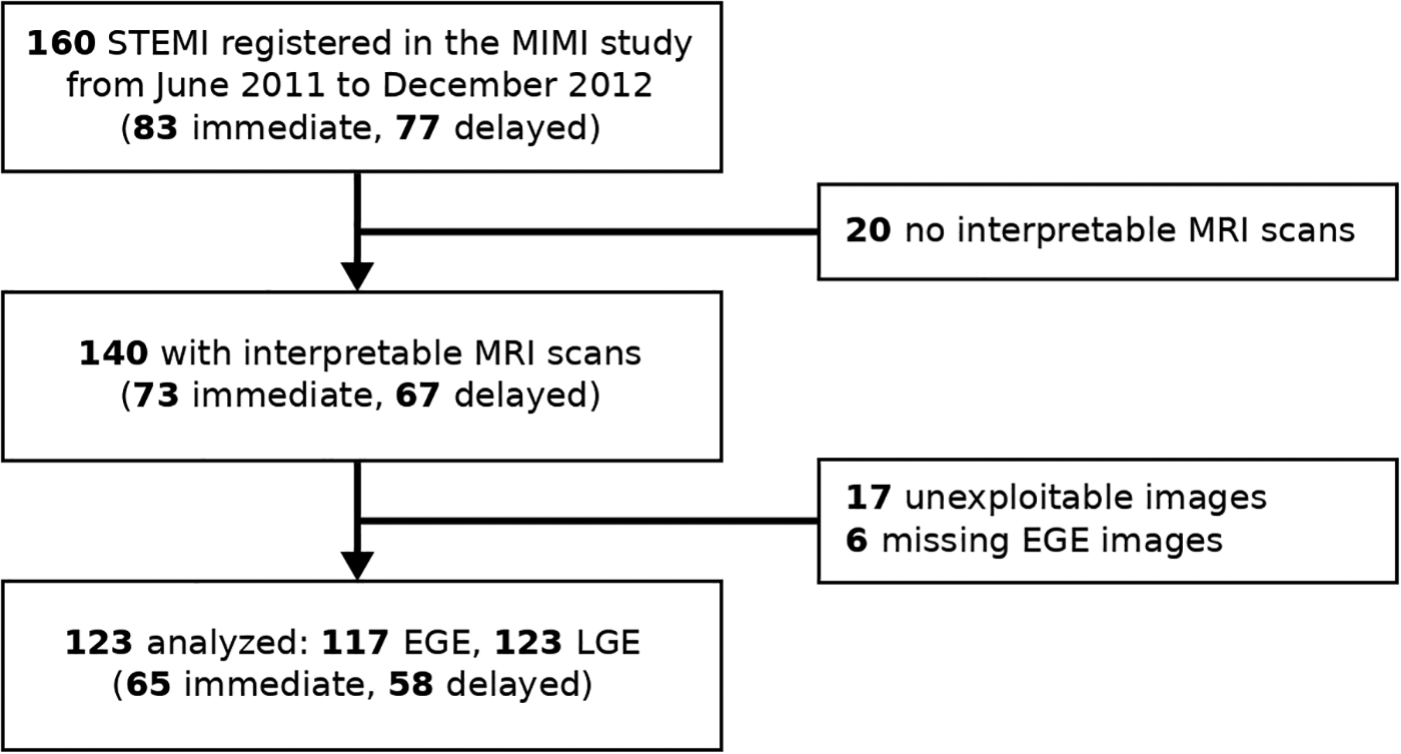
Study flow chart. EGE, early Gadolinium enhancement; LGE, late Gadolinium enhancement; STEMI, ST-elevation myocardial infarction.

The MRI protocol was performed in multiple centers 3 to 8 days after inclusion on 1.5 T systems. The analysis performed in this paper focused on EGE and LGE images for which LV and lesion segmentation was feasible (Figure 1), obtained according to the detailed MRI protocol provided in the Supplementary Material section of Belle et al. (10).

**Figure 2:**
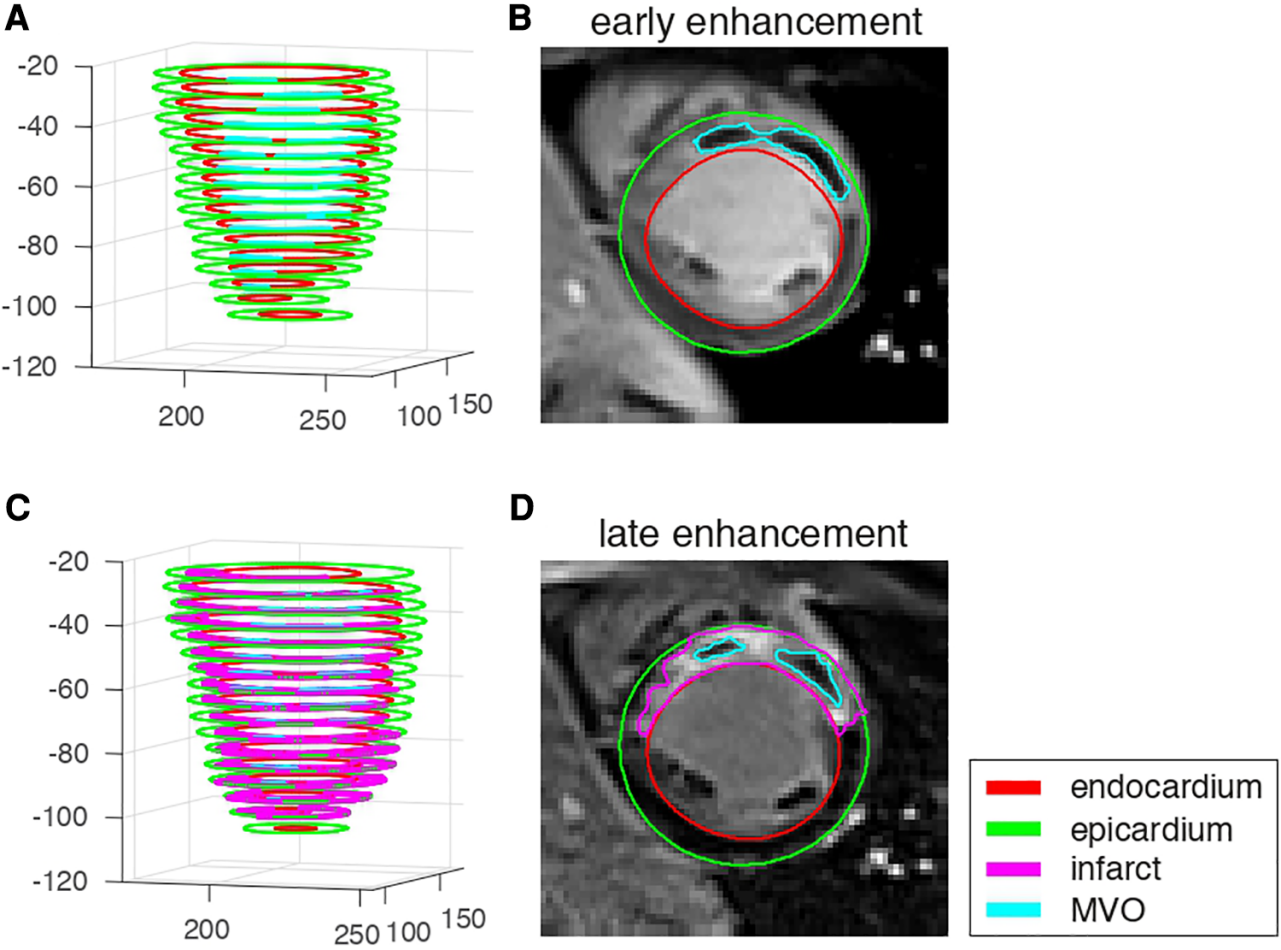
EGE and LGE data from one representative patient with microvascular obstruction and infarct. (**A,C**): 3D stacks of the segmented structures on each slice. (**B,D**): image data and segmented structures on a mid-cavity slice.

The myocardial and lesion contours were manually segmented offline by one experienced observer (LP) and controlled by two other experienced observers (MV, PCr) using commercial software (CVI42 v.5.1.0 Circle Cardiovascular Imaging, Calgary, Canada). They consisted of the endocardium, epicardium, infarct (from LGE), and early and late MVO (EGE and LGE, respectively) (Figure 2). The infarct zone was determined semi-automatically on LGE images using the full-width half-maximum method. MVO corresponded to hypo-enhancement within the infarct zone on the EGE and LGE images, and was segmented manually. The segmented images were exported as Dicom files. Contours were exported in the XML format as.cvi42wsx files. Both images and contours were loaded in Matlab (v.R2016a, MathWorks, Natick, USA) for post-processing and analysis.

The LV-RV junction was manually identified by one experienced observer (ND) using a single landmark on each slice. The myocardial borders around the LV outflow tract were marked by two landmarks to exclude this region on the concerned slices. Finally, the slice locations corresponding to the endocardial apex and the mitral level (the most extreme basal slice with more than 50% myocardium around the blood cavity) were identified. These locations were extrapolated in case the acquisition did not cover such slices.

### 2.3. Standardization onto a common geometry^1^

**Figure 3:**
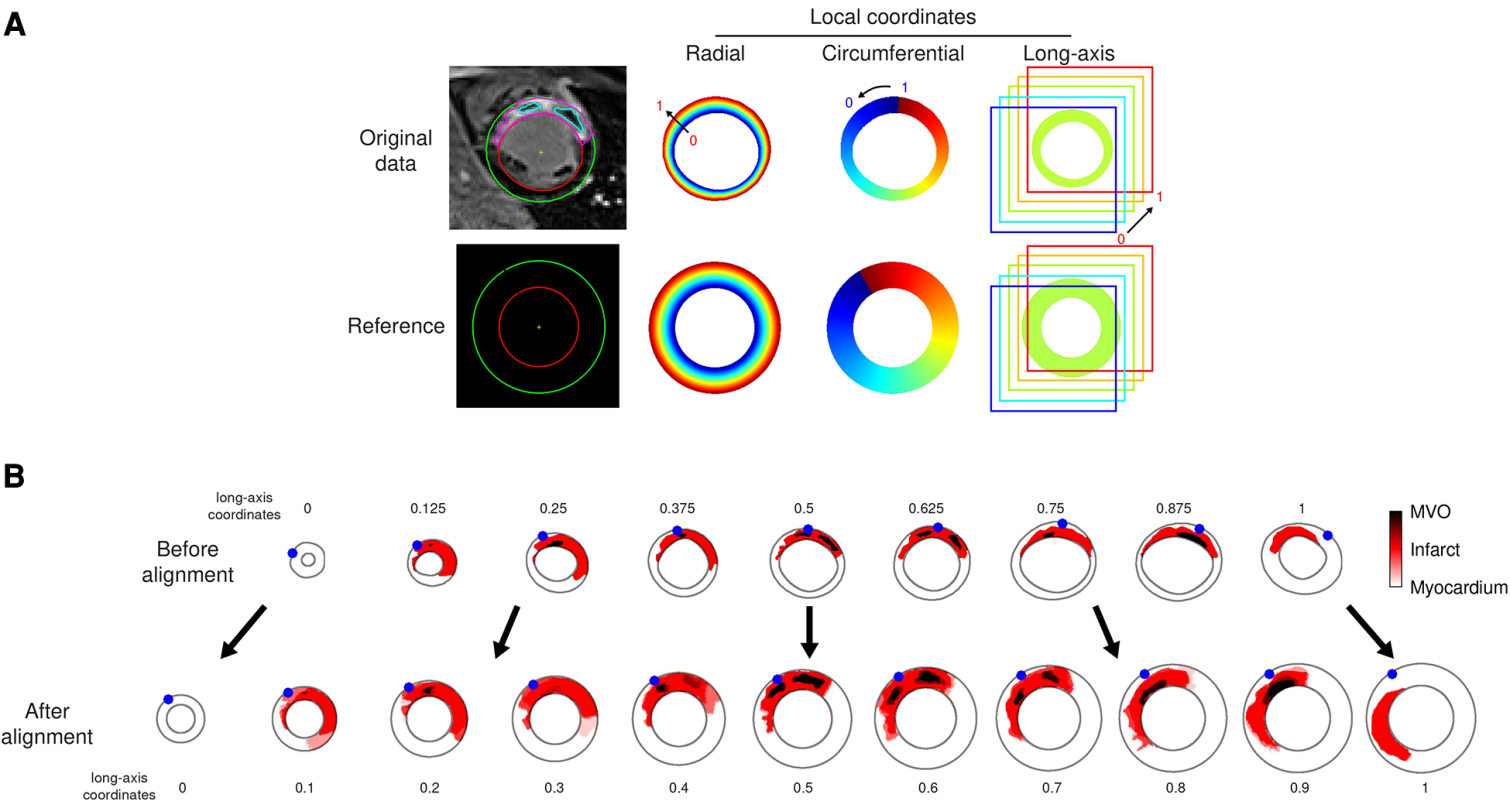
Data alignment steps illustrated on the representative patient from Figure 1. (**A**): automatically defined radial-circumferential-long axis coordinates on the studied subject’s anatomy, and on the reference used for the statistical analysis of the whole population. The color encodes the coordinates value ranging from 0 to 1 (long-axis coordinate = 0.625 for this slice). (**B**): standardization of the image data onto the reference (one slice out of two displayed for the sake of clarity, numbers indicate the standardized long-axis coordinates). The red and dark colors encode the segmented infarct and MVO, respectively. The blue dots stand for the location of the LV-RV junction on each slice.

We first parameterized the myocardium by defining radial, circumferential, and long-axis coordinates on each slice, for each individual. They were estimated on images oversampled by a factor 4 to prevent artifacts in case few pixels covered the myocardium, in particular along the radial direction. Their values ranged from 0 to 1, corresponding to endocardium-to-epicardium (radial), apex-to-base (long-axis), and the anticlockwise direction starting from the LV-RV junction (circumferential), as illustrated in Figure 3A, top row.

Then, we arbitrarily defined a common reference geometry onto which standardizing the image data, as a semi-ellipsoid with maximal endocardial and epicardial radii of respectively 30 and 50 pixels, represented on 21 slices of 80 × 80 pixels each (Figure 3A, bottom row).

Finally, images were warped by mapping each patient’s coordinates onto the reference coordinates. This was achieved by linear interpolation tailored for data defined on a scattered grid (here, the myocardial coordinates) (Figure 3B). No extrapolation was performed out of the minimal and maximal slices acquired.

### 2.4. Pixel-wise analysis

**Figure 4:**
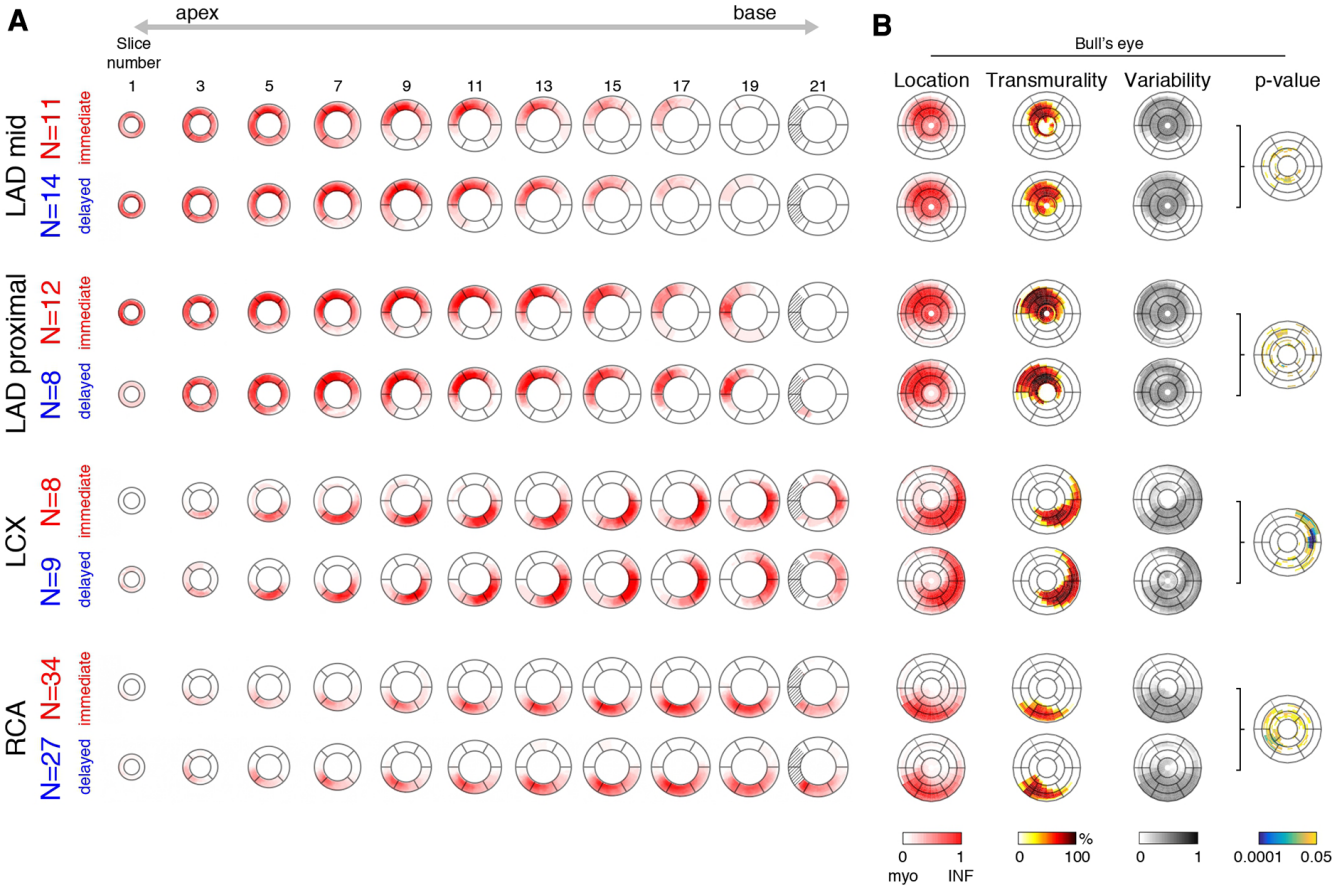
Representative infarct patterns depending on treatment (immediate vs. delayed) and territories (LAD mid or proximal, LCX, and RCA). (**A**): average pattern for each subgroup, from apex to base (one slice out of two displayed for the sake of clarity). The dashed region corresponds to incomplete myocardium at the basal level. (**B**): Bull’s eye representation that summarizes this information across slices: infarct location and variability, transmurality, and statistical differences between the two treatment options (*p*-value displayed in a logarithmic color scale).

Once standardized onto a common geometry, lesions can be compared at each pixel of the myocardium in the EGE and LGE images. We first estimated the representative (average) infarct pattern for each coronary territory and each treatment subgroup, and visualized it at each slice of the reference geometry (Figure 4A). A Bull’s eye representation also summarized this information across slices (Figure 4B). The Bull’s eye consisted of 21 concentric circles corresponding to all the slices of the reference geometry, and 24 segments around the circumference. The following information was displayed: (i) location, estimated as the maximal value of the infarct pattern along the radial direction; (ii) transmurality, estimated as the average value of the infarct pattern along the radial direction; and (iii) statistical differences between the two treatment options, assessed by the *p*-value from the Hotelling t-squared test (performed on the pixel map), displayed in a logarithmic color scale.

### 2.5. Link with other characteristics

Despite being standardized onto a common geometry, the infarct patterns still contain a lot of information, which is challenging to visualize and relate to other patient characteristics. We therefore estimated a simplified representation of all the individual subjects in the studied population that can be visualized in two dimensions. To do so, we used the t-SNE (T-distributed Stochastic Neighbor Embedding) algorithm (15), a widespread statistical method for reducing the dimensionality of complex data and visualizing it. Its purpose is similar to the very popular Principal Component Analysis algorithm, except that the latter performs linear dimensionality reduction, which is not fully suited to medical images compared to non-linear dimensionality reduction (16) as performed by t-SNE.

On our data, t-SNE provided a two-dimensional cloud of points where each point corresponds to a sample in the population, while forcing similar patterns to be represented by nearby points, and conversely dissimilar patterns to be represented by distant points. Then, we examined the link between infarct patterns and other variables of interest by coloring each point according to the value of the variable we inspect. We focused on a subset of relevant clinical and therapeutical variables related to patients’ risk: coronary artery of concern, ejection fraction, stenting strategy, delay from onset of symptoms to coronary intervention, sex, age, diabetic status, smoking status, and body mass index.

This was implemented in Python, using a public open-source version of t-SNE from the scikit-learn library^2^. The retained hyperparameters were two components (for two-dimensional visualization), perplexity of 10 (to define how much neighboring samples are considered for non-linear dimensionality reduction), and Principal Component Analysis initialization (for improved stability).

Before applying t-SNE, infarct patterns were realigned to the left anterior descending (LAD) territory, to better focus on the pattern shape without being confounded by different infarct localizations and analyse all the population at once. This was achieved by rotating all infarct patterns along the circumference such that the average infarct center of a given coronary territory is aligned to the average infarct center for the LAD territory, as previously reported (17).

### 2.6. Global descriptors

We also estimated commonly-used global descriptors of the main lesion characteristics: the proportion of damaged myocardium (for the infarct and the MVO, in %), the average transmurality over the infarcted myocardium (excluding the preserved myocardium, in %), and the endocardial surface area (in %). These characteristics were both estimated before and after standardization to the reference geometry to quantify the consistency of global observations regarding standardization (Table 1).

**Table 1:** Global characteristics of the lesion against the treatment subgroups, before and after standardization to a common reference geometry: proportion of lesion area compared to the whole myocardium (infarct and early/late MVO), infarct transmurality, and endocardial surface area compared to the whole myocardium.

	Before standardization			After standardization		
	Immediate stenting (*N* = 65)	Delayed stenting (*N* = 58)	*p*-value	Immediate stenting (*N* = 65)	Delayed stenting (*N* = 58)	*p*-value
Infarct area (% myocardium)						
LAD mid	20.8 (19.0–27.3)	24.4 (15.9–31.2)	0.767	18.2 (11.6–23.4)	18.8 (11.1–23.4)	1.000
LAD proximal	30.3 (25.6–36.6)	26.8 (20.9–37.8)	0.571	23.1 (19.1–25.9)	21.5 (16.5–30.6)	0.792
LCX	21.1 (16.3–29.9)	19.8 (18.0–28.1)	1.000	18.7 (14.9–21.9)	18.4 (16.6–26.7)	0.481
RCA	12.0 (9.5–19.3)	13.3 (10.4–22.1)	0.412	12.3 (8.1–18.0)	11.9 (9.4–20.1)	0.611
Early MVO area (% myocardium)						
LAD mid	2.0 (0.1–4.7)	1.3 (0.1–4.7)	0.976	0.8 (0.0–2.4)	0.5 (0.0–3.8)	0.976
LAD proximal	2.5 (0.9–9.6)	5.3 (3.7–9.5)	0.270	1.5 (0.5–7.8)	4.5 (2.3–7.3)	0.305
LCX	6.9 (0.2–9.7)	3.8 (1.4–7.9)	0.779	5.5 (0.1–8.0)	3.2 (1.1–6.6)	0.779
RCA	0.6 (0.0–3.7)	2.1 (0.0–4.3)	0.466	0.3 (0.0–3.0)	1.1 (0.0–3.8)	0.470
Late MVO area (% myocardium)						
LAD mid	0.1 (0.0–3.0)	1.1 (0.0–3.8)	0.572	0.1 (0.0–2.6)	0.8 (0.0–2.8)	0.501
LAD proximal	1.8 (0.4–9.3)	4.1 (1.4–7.7)	0.305	1.1 (0.2–8.1)	2.9 (0.9–6.7)	0.384
LCX	3.7 (0.0–6.1)	2.7 (0.8–8.8)	0.481	3.6 (0.0–5.3)	2.3 (0.7–8.0)	0.743
RCA	0.0 (0.0–0.9)	0.6 (0.0–2.5)	**0**.**044**	0.0 (0.0–1.0)	0.4 (0.0–2.1)	0.065
Transmurality (%)						
LAD mid	62.4 (50.4–81.3)	61.7 (51.3–68.2)	0.893	55.8 (51.0–75.0)	58.5 (49.4–64.4)	0.687
LAD proximal	69.8 (61.0–76.3)	69.3 (60.5–76.1)	0.910	69.0 (59.4–73.3)	66.5 (56.5–74.4)	0.792
LCX	62.0 (52.2–73.8)	69.1 (56.7–76.0)	0.321	56.5 (44.9–66.8)	64.0 (51.1–66.5)	0.481
RCA	55.6 (46.7–66.4)	63.4 (45.5–74.2)	0.184	50.6 (40.2–60.3)	57.0 (36.5–66.6)	0.182
Endocardial surface area (% endocardium)						
LAD mid	25.3 (20.0–36.6)	28.2 (20.9–38.5)	0.687	24.7 (19.0–29.3)	29.8 (20.5–35.3)	0.467
LAD proximal	37.8 (27.7–41.5)	34.2 (27.6–45.4)	1.000	33.2 (30.7–38.7)	32.2 (23.5–44.6)	0.970
LCX	25.6 (15.1–35.8)	24.5 (17.4–27.6)	0.673	25.2 (19.4–34.3)	26.6 (20.7–30.3)	0.963
RCA	13.5 (8.0–19.0)	12.8 (8.4–22.5)	0.674	14.4 (8.4–21.8)	12.1 (10.2–21.8)	0.850

MVO, micro-vascular obstruction; LAD, left anterior descending; LCX, left circumflex; RCA, right coronary artery. Bold font highglights statistically significant differences.

### 2.7. Reproducibility analysis

The identification of the LV-RV junction on each slice, and the identification of the apex and mitral valve slice locations were repeated by the same operator and another experienced operator (LP). The reproducibility of these measurements (Supplementary Figures S1, S2) and its effect on the statistical analysis of the standardized lesion patterns (Supplementary Figure S3) is reported in Supplementary Material.

### 2.8. Statistical analysis

Standard statistical analysis was performed in complement of the pixel-wise analyses exposed in the previous sections, as follows. Continuous variables were expressed as median and interquartile range, with Mann-Whitney U-test used for inter-groups comparisons. Categorical variables were expressed in percentage over the number of samples, with Fisher’s exact test used for inter-groups comparisons. *P*-values below 0.05 were considered statistically significant. All the patient and the global lesion characteristics were analyzed using the SPSS software (Version 21.0, IBM Corp, Armonk, NY).

## 3. Results

### 3.1. Patient characteristics

The original MIMI study enrolled 160 patients between June 2011 and December 2012, of which 140 patients had interpretable MRI scans (10). In the present study, the datasets of 17 patients were unexploitable with our analysis techniques for technical issues (sub-optimal LV coverage, or image artifacts); the EGE images were not analyzed for these 17 patients and 6 additional patients for which the EGE data were missing. We therefore analyzed the EGE and LGE images for 117 and 123 patients, respectively, corresponding to 65 patients with immediate stenting and 58 patients with delayed stenting (Figure 1). The baseline characteristics of the patients are summarized in Table 2.

**Table 2:** Main clinical and lesion characteristics depending on the treatment subgroup.

	Immediate stenting (*N* = 65)	Delayed stenting (*N* = 58)	*p*-value
Age (years)	55.9 (47.9–65.0)	60.7 (50.4–68.7)	0.095
Sex (% male)	55 (84.6)	43 (74.1)	0.181
Body mass index (kg/m^2^)	26.5 (23.78–29.4)	26.0 (23.6–28.6)	0.413
EDV (ml)	153 (136–186)	152 (124–176)	0.221
ESV (ml)	73 (59–91)	73 (56–90)	0.914
LVEF (%)	54 (47–62)	51 (46–60)	0.256
Infarct (g)	30.38 (14.03–44.67)	27.89 (16.65–45.11)	0.905
Infarct (% of LV mass)	18.64 (8.88–24.78)	20.75 (12.43–27.60)	0.361
MVO (g)	2.00 (0.00–8.41)	5.27 (0.53–10.31)	0.134
MVO (% of LV mass)	1.29 (0.00–4.43)	3.86 (0.35–6.45)	0.059
Infarct artery location			
LAD mid	11	14	1.000
LAD proximal	12	8	0.642
LCX	8	9	0.206
RCA	34	27	0.224

EDV, end-diastolic volume; ESV, end-systolic volume; LVEF, left ventricular ejection fraction; MVO, micro-vascular obstruction; LAD, left anterior descending; LCX, left circumflex; RCA, right coronary artery.

The median resolution of the analyzed images was 1.5625 × 1.5625 × 5 mm. The LV ranged over 17 ± 2 slices, with 1 ± 1 extra slices lying out of the acquired stack of slices.

### 3.2. Pixel-wise analysis

Figure 4 displays the representative infarct patterns associated to each territory and treatment. The left side shows these patterns slice by slice from apex to base, while the right side uses Bull’s eye views to summarize specific characteristics of the patterns across slices.

Infarct patterns (see “location”, “transmurality” and “variability” Bull’s eye plots for a synthetic view) were roughly comparable between the two treatments for all territories and across all slices. However, for the left circumflex (LCX) infarcts, higher transmurality was observed with delayed stenting at the lateral locations (average transmurality in these segments: 36% (immediate) vs. 53% (delayed)). A similar trend was observed for the right coronary artery (RCA) infarcts at the inferior/inferoseptal locations (average transmurality in these segments: 32% (immediate) vs. 38% (delayed)). These subtle observations were confirmed by the “*p*-value” Bull’s eye, which quantified statistical differences between the two treatment groups at each location. The LAD mid and LAD proximal groups respectively had 9% and 13% of myocardial locations with a *p*-value <0.05, but rather sparsely distributed across the ventricle and not necessarily matching the infarct zone. In contrast, the LCX and RCA groups respectively had 15% and 23% of myocardial locations with a *p*-value <0.05, including marked statistical differences grouped over a portion of the infarct zone.

Early MVO was absent in 28/117 patients: 16/61 (28%) with immediate stenting and 12/56 (23%) with delayed stenting. Late MVO was absent in 48/123 patients: 32/65 (49%) with immediate stenting and 16/58 (28%) with delayed stenting. No infarct was observed in one patient (immediate stenting, RCA ischemia).

### 3.3. Link with other characteristics

**Figure 5:**
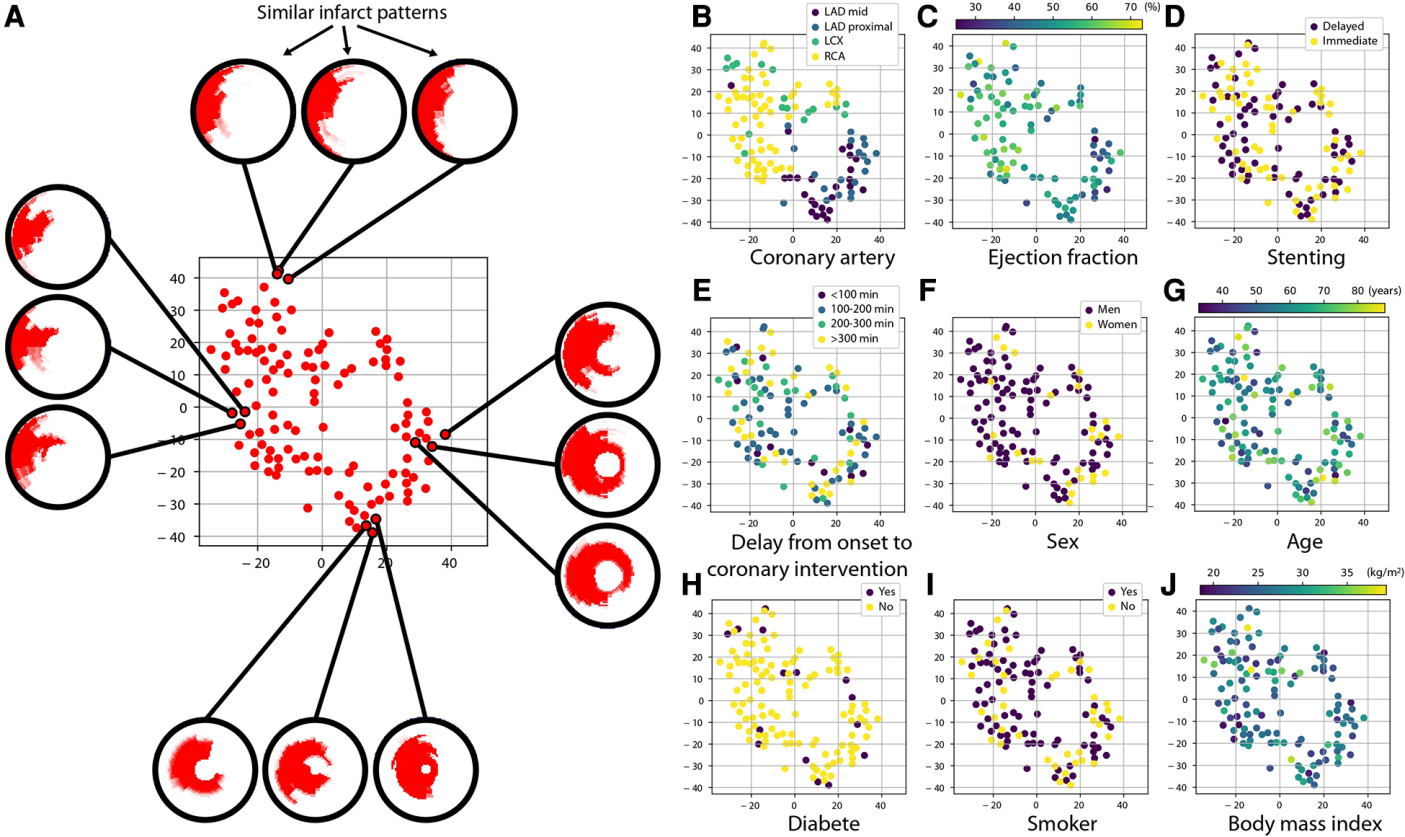
Link between infarct patterns and other variables of interest. Each subplot depicts the same cloud of points, which is a simplified two-dimensional visualization (of arbitrary units) obtained with the t-SNE algorithm of how subjects differ regarding their infarct shape: two subjects with similar/dissimilar infarct shape are placed close/far from each other, as illustrated on subplot (**A**). Beforehand, infarct patterns of any territory were realigned to match the LAD territory, so that observations focus on the actual pattern shape and not its position around the myocardium. On subplots (**B**–**J**), the color code indicates the value of a specific clinical and therapeutic characteristic of the patients. Infarct shapes appear consistent across a given coronary artery territory and differ more between different territories. A slightly similar trend is observed regarding ejection fraction. In contrast, no link is observed for the other variables.

Figure 5 proposes a two-dimensional visualization of the whole population where subjects with similar/dissimilar infarct shapes are mapped close/far from each other, as estimated by the t-SNE dimensionality reduction algorithm. On each subplot, the color code corresponds to the values of a specific clinical or therapeutic variable for each subject, to examine its potential link with the infarct patterns. We remind that to construct this plot, infarct patterns of any territory were realigned to match the LAD territory, so that observations focus on the actual pattern shape and not its position around the myocardium.

We first observe that even when this anatomical alignment was performed, the shapes of infarct patterns showed anatomical consistency across subjects from the same coronary artery. The shapes of LAD infarcts were quite similar, while RCA and LCX infarcts showed more dissimilar shapes that are likely related to broader anatomical and collaterality variations across patients having RCA and LCX culprit arteries. A more subtle grouping of patterns was observed regarding ejection fraction, which might reflect the varying impact of each coronary artery on the cardiac function. In contrast, no specific grouping of patterns was observed for the stenting strategy, confirming the main finding of the MIMI study. The lack of grouping of patterns for all the other selected variables (delay from onset of symptoms to coronary intervention, sex, age, diabetic status, smoking status, body mass index) indicated the lack of bias and the quality of randomization.

Similar observations were obtained by multiple launches of the t-SNE algorithm, which confirmed results against the random state intrinsic to this algorithm.

### 3.4. Global descriptors

Table 2 examines the five global descriptors of the lesion patterns (area of the infarct, early MVO, and late MVO, transmurality, and endocardial surface area). No statistically significant differences were observed between the two treatments, for all territories, for all-except-one measurements before standardization / for all after standardization to the common reference geometry.

### 3.5. Reproducibility analysis

Supplementary Figure S1 summarizes the intra- and inter-operator variability in the identification of the LV-RV junction on each slice, for the EGE and LGE images. Angles were defined by the lines joining the measurement of each operator and the center of the LV cavity, on each slice. Intra-operator differences were low, with higher differences near the apex but variability in the range [−20.0°,8.7°]. Inter-operator differences were higher but moderate, with higher differences near the apex but variability in the range [−18.9°,23.4°]. Observations were comparable for the EGE and LGE images.

Supplementary Figure S2 complements this analysis by summarizing the intra- and inter-operator variability in the identification of the apical and basal slices, for the EGE and LGE images. Differences were low, mostly ±1 slice both for the EGE and LGE images. They were higher for the intra-operator experiment, in particular at the basal level (more cases with a difference of ±2 slices, a few up to ±3 slices), in part due to the difficulty of extrapolating this location when the LV was not fully covered by the slices.

Finally, Supplementary Figure S3 provides a display similar to the Bull’s eyes from Figure 4 for the location, transmurality, and variability of the infarct patterns, and for the *p*-value quantifying statistical differences between the two treatment groups, for identification of the LV-RV junction and basal/apical slices repeated by the same operator or by a second one. Patterns are very similar to the ones in Figure 4, and lead to equal conclusions: comparable patterns between the two treatments for all territories and across all slices, but higher transmurality with delayed stenting for the LCX and RCA infarcts, confirmed by statistically significant differences near the infarct zone, contrary to the LAD mid and LAD proximal infarcts.

## 4. Discussion

We proposed a fresh look at the characterization of myocardial lesions (infarct and early/late MVO) to go beyond global assessment with quantitative and standardized comparisons up to the pixel resolution. On the delayed enhancement imaging data from the MIMI study, which aimed at assessing acute myocardial infarction patients while comparing different reperfusion strategies, our results not only confirmed at a much finer resolution and in a more standardized manner that immediate and delayed stenting led to comparable infarct and reperfusion lesions, but revealed a deleterious effect of delayed stenting at specific locations. They also indicated that the infarct shape was not related to other clinical and therapeutic variables of interest.

Comparing delayed vs. immediate stenting highly depends on the chosen MRI endpoints (2). In the original MIMI study (10), the primary endpoint was MVO (% of total LV mass observed by MRI 5 days after inclusion). Primary endpoints were slightly different in similar randomized controlled trials of moderate sample sizes. The INNOVATION study used the infarct size (% of total LV mass observed by MRI 30 days after inclusion) (14), while the DEFER-STEMI study considered the incidence of no-/slow-reflow (Thrombolysis In Myocardial Infarction ≤2, 2 days after inclusion) (13). They concluded that delayed stenting may better preserve the myocardium, not necessarily for all territories or supported by statistically significant differences. The much larger DEFER-DAMANI randomized controlled trial did not find evidence of improvement with delayed stenting using a composite score of clinical outcomes (18). Its findings were confirmed by a sub-study, which considered the final infarct size as primary endpoint (19). Current guidelines do not therefore recommend delayed stenting (12). Nonetheless, differences in the conclusions brought by these studies should be tempered, mainly due to different primary endpoints, different delays in the stenting procedure, and different spectrum of included patients (11). Delayed stenting might still benefit a subset of patients, to be confirmed by other randomized controlled trials and re-analysis of the existing studies as targeted in the PRIMACY trial (20).

In the literature, the lack of clear differences between treatment options may also come from the simplicity of global descriptors (occurrence, size, transmurality, or endocardial surface area as a surrogate of the area at risk, mainly) to assess lesions of complex shapes. Many studies investigating the cardiac function, not necessarily with MRI, already underlined the value of regional or local descriptors of the observed diseases (7, 16). Our approach also goes into this direction and provides a more complete and standardized assessment of the lesion patterns up to each pixel of the myocardium. In our study, this local quantitative assessment not only confirmed the lack of benefit from delayed stenting, but also revealed subtle differences insufficiently rendered by global measurements and suggesting a deleterious effect visible at some locations (in particular, higher transmurality with delayed stenting for the LCX and RCA).

Statistical atlases have been widely used to quantify shape differences across of a population (8), as recently demonstrated on acute myocardial infarction patients (9). In our work, we used these techniques beyond shape assessment and analyzed the imaging data on a common geometry. This standardization was inspired by previous works on the statistical analysis of myocardial infarct (21, 22), except that these considered 3D mesh data (while we directly operated on the image data), and did not incorporate MVO. The use of such spatial alignment goes in the sense of recent recommendations, to better standardize the imaging data (2).

### 4.1. Study limitations

Seventeen patients were not analyzed due to technical issues related to the image quality. Besides, our population size was moderate, and the statistical analysis of lesion patterns may be affected by this sample size, in particular in case of small or unbalanced subgroups as observed for some territories. We therefore supported as much as possible our observations with complementary measurements (the variability and statistical differences computed locally, the global lesion characteristics, and intra/inter-operator reproducibility analysis).

Spatial alignment required the semi-automatic segmentation of the myocardium and the manual identification of few landmarks on each slice, which is time-consuming and needs careful quality control in the current implementation. This could be solved by considering recent advances in the automatic segmentation of delayed enhancement MRI, when its accuracy reaches acceptable ranges on all slices on routine imaging data (23, 24).

Non-linear statistical analysis techniques may overcome some limitations of the techniques we used here to estimate representative lesion patterns (21, 25), but would require larger populations and may still provide limited rendering of sharp transitions in the analyzed data, such as the interface between the myocardium and the infarct (17).

## 5. Conclusion

Our methodology allows assessing the acute myocardial infarction lesions much beyond commonly used global descriptors while delivering a synthetic picture of a population, and could be used for standardized reporting in many cardiac MRI clinical research studies. This approach provides quantitative finer insights into how lesions differ between subgroups of subjects, and enables to locate a given subject within the population/group response in a logic of personalized medicine. On the MIMI study, it confirmed at a much finer scale the comparable myocardial damage between immediate and delayed stenting, and even suggested a deleterious effect of delayed stenting visible at some locations. This strategy appears highly promising to analyze in a much more integrated manner the multi-parametric and longitudinal data from ischemia-reperfusion studies, and better state on potential therapies to reduce myocardial loss after myocardial infarction.

## Data Availability

Data generated or analyzed during the study are not publicly available. Requests to access these datasets should be directed to nicolas.duchateau@creatis.insa-lyon.fr.

## Footnotes

^1^Code and demo data corresponding to this standardization process will be made accessible upon acceptance at: https://github.com/nicolasduchateau/CMR-imageDataAlignment

^2^ https://scikit-learn.org/stable/modules/generated/sklearn.manifold.TSNE.html

## Supplementary material

SUPPLEMENTARY FIGURE S1

Intra- and inter-operator reproducibility in the identification of the LV-RV junction, on all slices of EGE and LGE images.

SUPPLEMENTARY FIGURE S2

Intra- and inter-operator reproducibility in the identification of the apex and mitral valve slice locations, on EGE and LGE images.

SUPPLEMENTARY FIGURE S3

Representative infarct patterns depending on treatment (immediate vs. delayed) and territories (LAD mid or proximal, LCX, and RCA), when the standardization process was repeated (a) for the same operator or (b) performed by a second operator. Bull’s eye representation only, display similar to Figure 4.
