# Supplementary figures and images for: Pixel-wise statistical analysis of myocardial injury in STEMI patients with delayed enhancement MRI
Source: Front Cardiovasc Med. 2023 Jun 16;10:1136760. doi: 10.3389/fcvm.2023.1136760 (PMC10313104; doi:10.3389/fcvm.2023.1136760)

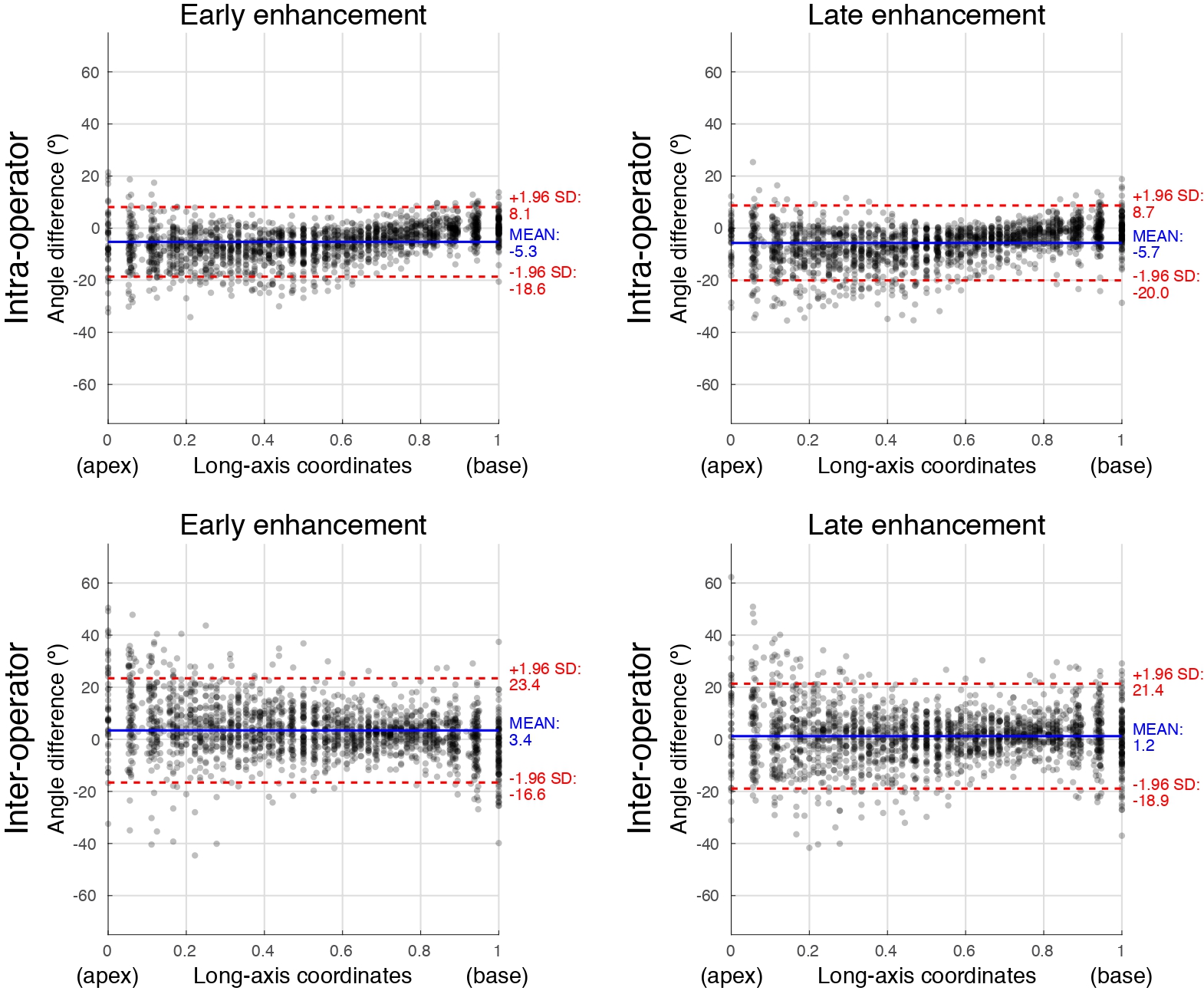

Supplement: Supplementary file 1 [file Image1.jpeg]

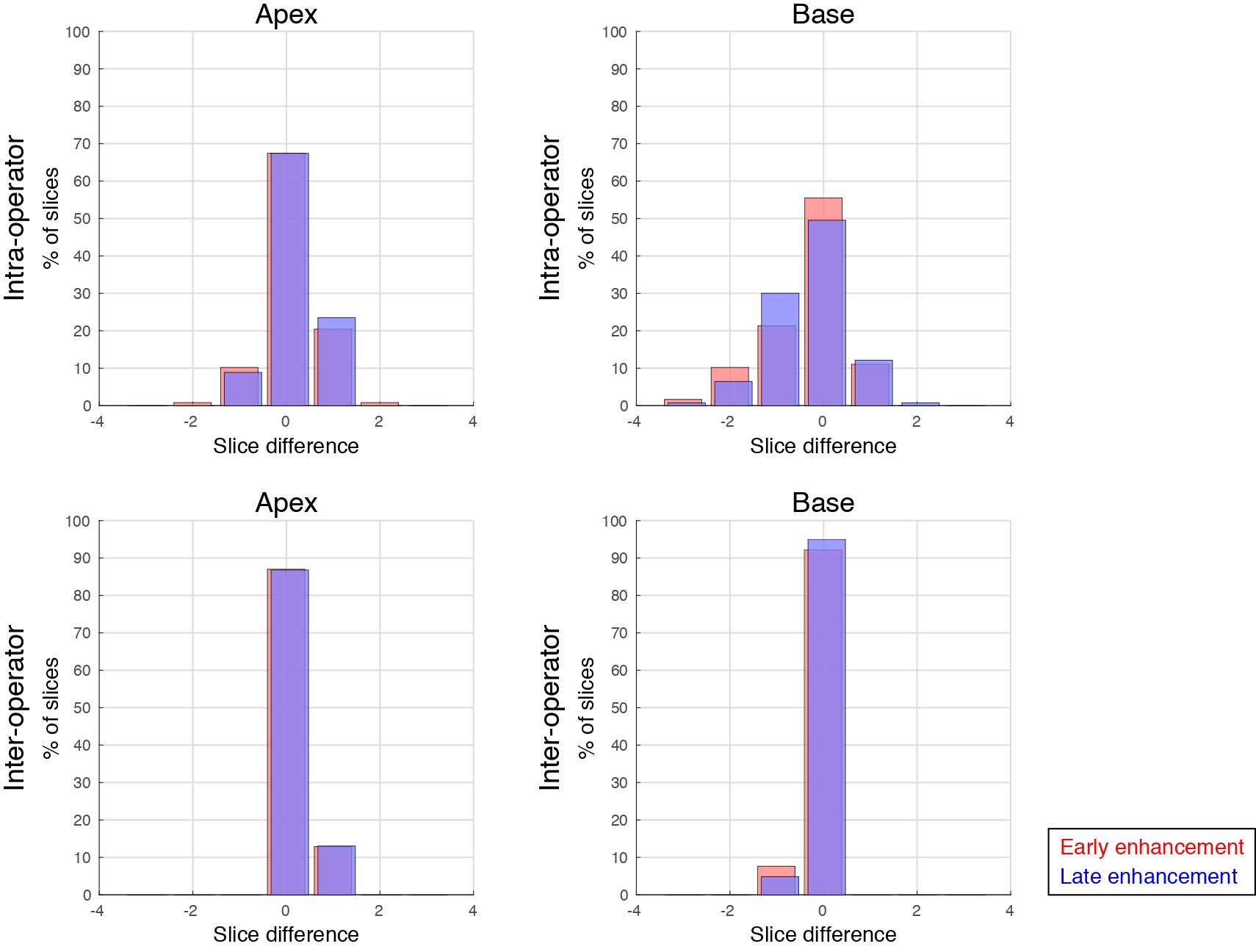

Supplement: Supplementary file 2 [file Image2.jpeg]

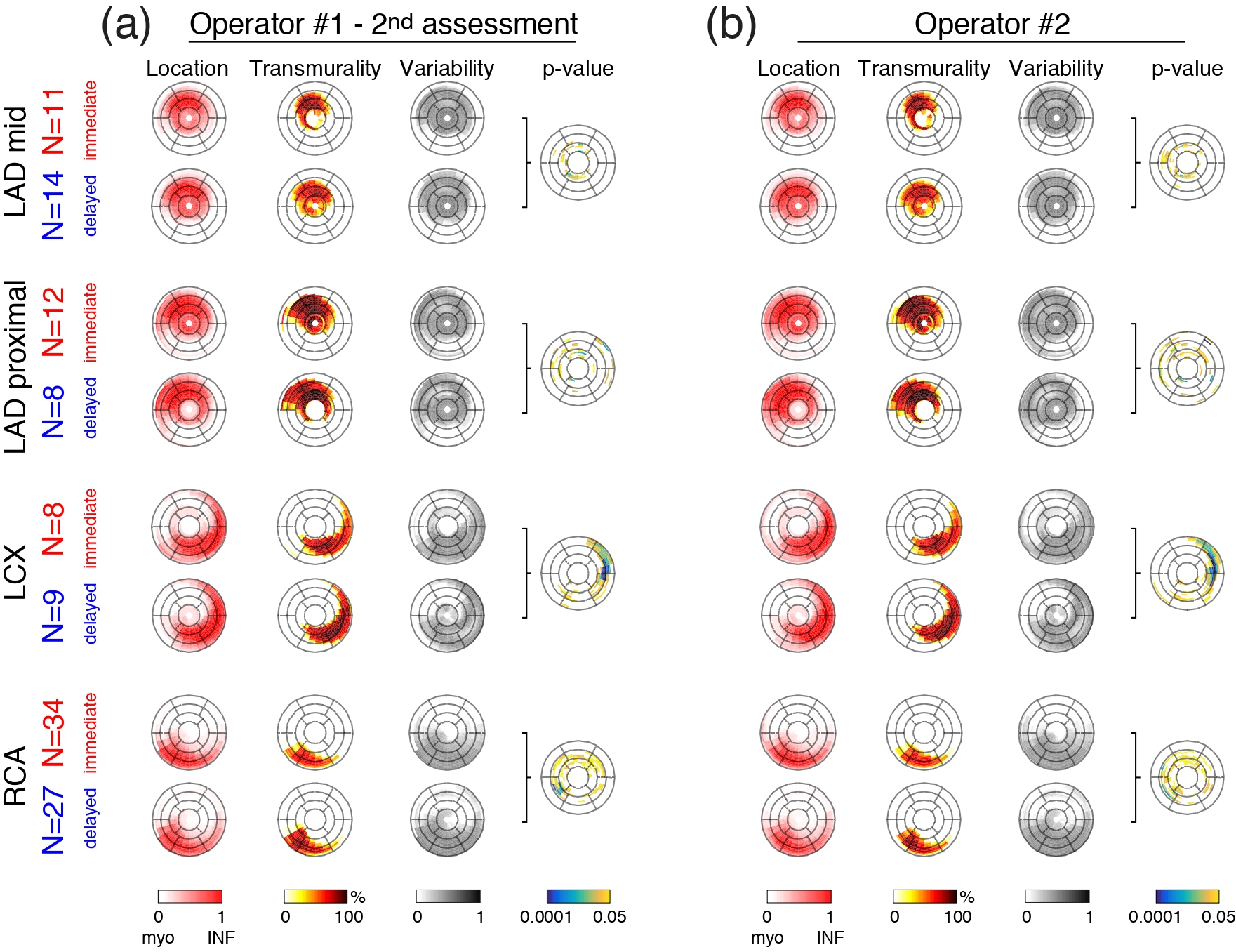

Supplement: Supplementary file 3 [file Image3.jpeg]
